# Gout Versus Pseudogout in the Medically Underserved Rio Grande Valley

**DOI:** 10.7759/cureus.79457

**Published:** 2025-02-22

**Authors:** Blake C Martin, Fernando Cisneros, Kristina Vatcheva, Michael D Sander

**Affiliations:** 1 Department of Medical Education, University of Texas Rio Grande Valley School of Medicine, Edinburg, USA; 2 Department of Biostatistics and Epidemiology, University of Texas Rio Grande Valley School of Medicine, Edinburg, USA; 3 Department of Orthopedic Surgery, University of Texas Rio Grande Valley School of Medicine, Edinburg, USA

**Keywords:** bone and joint, gout, joint disease, joint inflammation, pseudogout

## Abstract

Background

Gout and pseudogout are inflammatory joint conditions, with gout being one of the most prevalent etiologies of chronic inflammatory arthritis in the United States. The patient population in the Rio Grande Valley (RGV) has a distinct demographic profile that warrants the exploration of various health conditions. The primary objective of this study was to expand the knowledge of orthopedics and determine if there were demographic disparities between gout and pseudogout in this community. We hypothesized that there would be demographic disparities between gout and pseudogout depending on age, sex, and ethnicity.

Methods

This was a retrospective chart review, and data was gathered from the University of Texas Rio Grande Valley (UTRGV) UTHealth electronic database from January 1, 2017, to January 1, 2024. We collected and analyzed medical charts of individuals who were diagnosed with gout or pseudogout using the International Classification of Diseases version 10 (ICD-10) diagnosis codes M10 for gout and M11.2 for pseudogout. Patients’ characteristics were described by gout status using frequency (n) and percentage (%) for categorical variables.

Results

Individuals in age groups 40-65 years and greater than or equal to 65 years compared to patients aged less than 40 years old had significantly higher odds of having gout versus no gout. Women compared to men had 88% lower odds of having gout compared to no gout (odds ratio {OR}=0.12; 95% confidence interval {CI}: 0.08, 0.17; p<0.0001) and 83% lower odds of having gout compared to pseudogout (OR=0.17; 95% CI: 0.08, 0.39; p<0.0001). Hispanic or Latino ethnicity, compared to non-Hispanic and non-Latino ethnicity, had significantly lower odds of gout (OR=0.32; 95% CI: 0.23, 0.45; p<0.0001) and pseudogout (OR=0.30; 95% CI: 0.14, 0.65; p<0.0001) compared to no gout. Individuals classified as overweight or obese compared to normal-BMI patients had significantly higher odds of having gout compared to patients with no gout. Marital status was the only difference between univariable and multivariable analysis and was not significantly associated with gout status according to the multivariable model.

Conclusion

Individuals of various demographics in the underserved RGV community, and possibly demographically similar communities, may be at increased risk for gout or pseudogout. This warrants further research on these conditions in this region to further improve the knowledge and possibly prevent the numerous effects of these conditions on an individual’s quality of life.

## Introduction

Gout

Gout is one of the most prevalent etiologies of chronic inflammatory arthritis in the United States and is characterized by the deposition of monosodium urate monohydrate crystals, the end product of human purine metabolism, in joints and tissues [1]. Although it predominantly affects the first metatarsophalangeal joint, it may occur in various other joints [1]. This condition may manifest in various ways, such as an acute gout flare, chronic gouty arthropathy, the accumulation of urate crystals in the form of tophaceous deposits, uric acid nephrolithiasis, or chronic nephropathy [1]. Generally, gout is multifactorial with genetics, medical comorbidities, and dietary factors playing an important role [1]. Hyperuricemia is arguably the most important risk factor, although a protein-rich diet, obesity, older age, male sex, alcohol consumption, comorbid diseases, certain medications, and genetic predisposition may also contribute to the development of gout [1,2]. However, although rare, a single genetic defect may be solely responsible for causing gout [1]. Generally, the prevalence of gout is 1%-4%; however, the prevalence of gout varies by age, sex, and country of origin [1]. In general, men are affected greater than women [1,3,4]. In Western nations such as the United States, the prevalence is significantly higher in men (3%-6%) compared to women (1%-2%), showing a possible sixfold difference between sexes [1]. African-American individuals have an increased prevalence compared to White individuals in the United States [1]. Increased age is also associated with an increase in prevalence, although plateauing after 70 years of age [1]. Regarding incidence rates, gout has displayed an increase over the past several decades, with a greater increase observed in men than in women and rising with age [1].

Pseudogout

Pseudogout, formally called calcium pyrophosphate deposition disease, is similar to gout in that it is characterized by the deposition of crystals in joints involving the synovial and periarticular tissues [5]. However, the crystals in pseudogout are made of calcium pyrophosphate dihydrate and typically affect larger weight-bearing joints such as the knee [5]. It can present as an acute flare or chronically with a waxing and waning clinical course that may last for several months [5]. Many individuals with pseudogout present with an underlying joint disease or metabolic abnormalities predisposing to calcium pyrophosphate dihydrate deposition [5]. The current literature shows that hyperparathyroidism has the highest positive association with pseudogout, followed by gout, osteoarthritis, rheumatoid arthritis, and hemochromatosis [5,6]. Osteoporosis, hypomagnesemia, chronic kidney disease, and calcium supplementation are other comorbidities that have also been associated with pseudogout [5]. This condition most commonly affects individuals over the age of 65 with 30%-50% of said individuals being over the age of 85 years [5,6]. It is rare for this condition to present in an individual under the age of 60 [2]. One cross-sectional study of US veterans reported a point prevalence of 5.2 per 1000, with an average age of 68 years and 95% male prevalence [5,7]. Another large cross-sectional study reported a 4% crude prevalence of pseudogout in the general population [5,7].

Study significance and aim

The patient population in the Rio Grande Valley (RGV) has a distinct demographic profile that warrants the exploration of various health conditions. This population has an increased prevalence of chronic conditions, such as obesity and diabetes [8]. Along with these chronic conditions, the community is medically underserved and impoverished and hosts a large population of undocumented individuals [9,10]. The Latino construct of machismo is another factor that may contribute to the health of this region as it may prevent Hispanic men from seeking medical help due to it being perceived as feminine [11]. To our knowledge, there is no study that has compared these two similar conditions in this unique population. The primary objective of this study was to expand the knowledge of orthopedics and determine if there were demographic disparities between gout and pseudogout in this community. We hypothesized that there would be demographic disparities between gout and pseudogout depending on age, sex, and ethnicity.

## Materials and methods

Study design and data collection

This was a retrospective chart review, and institutional review board (IRB) approval was obtained prior to starting this study. Data was gathered from the University of Texas Rio Grande Valley (UTRGV) UTHealth electronic database. Data collection included medical charts from January 1, 2017, to January 1, 2024. We collected and analyzed medical charts of individuals who were diagnosed with gout or pseudogout. These conditions were obtained by using the International Classification of Diseases version 10 (ICD-10) diagnosis codes M10 for gout and M11.2 for pseudogout. For each individual, various demographics were collected, including BMI, sex, age at diagnosis, race/ethnicity, and marital status.

Inclusion and exclusion criteria

Individuals over the age of 85 were included as a group indicated as “>85” years of age. Individuals who were not seen by a UTRGV-associated institution were not included in the study. If there were duplications of an individual’s medical chart, such as an individual having more than one appointment, the earliest date, the date the patient was diagnosed with gout or pseudogout, was included. If a patient was diagnosed with more than one of the conditions analyzed, all disorders were included and analyzed based on the date of diagnosis and the demographics of the patient at the point in time of that specific diagnosis. Individuals with complete data (having all the analyzed patient characteristics) were included during the univariate and multivariate statistical analyses.

Data analysis

Patients’ characteristics were described by gout status using frequency (n) and percentage (%) for categorical variables. Univariable and multiple multinomial logistic regression models were used to evaluate the association between gout status and socio-demographic characteristics. Crude and adjusted odds ratios (ORs) and their respective 95% confidence intervals (CIs) were estimated. All statistical tests were two-sided and were performed at a significance level of 0.05. All statistical analyses were conducted using Statistical Analysis System (SAS) 9.4 (SAS Institute Inc., Cary, NC).

## Results

Gout proportion in analyzed patients

**Figure 1:**
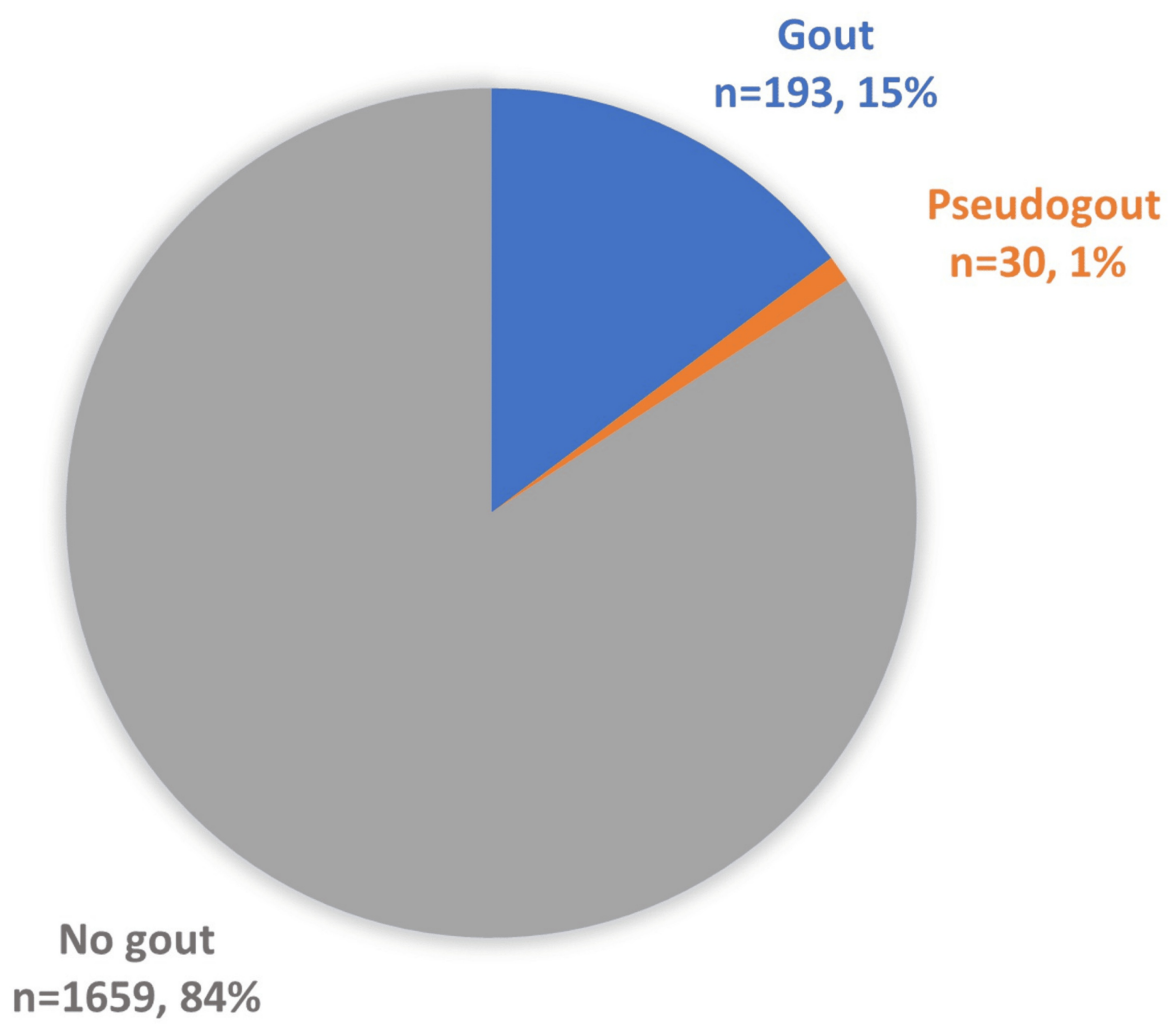
Proportion of patients with gout, pseudogout, or neither (no gout). The data has been presented in raw totals and percentages.

In total, 4413 patients from UTRGV-associated institutions were used to calculate the proportions of gout and pseudogout. Around 15% (n=651) of the patients had gout, and 1% (n=45) had pseudogout (Figure 1).

Characteristics of the study population

From the extracted 4413 patient records (Table 1), a subset of 1882 patients (Table 2) with complete data were used in the statistical analysis. Table 1 provides the main socio-demographic patient characteristics for all the patient records. The majority of the patients in this study were between the ages 40 and 65 (n=1778, 40.29%), women (n=2509, 56.85%), and of Hispanic or Latino ethnicity (n=3356, 85.61%). Slightly, more individuals were married or had a partner (n=1808, 51.73%) compared to individuals who were single, divorced, or widowed (n=1687, 48.27%). In terms of BMI, the majority of individuals were obese (n=1220, 53.56%), followed by overweight (n=608, 26.69%), and the minority were of normal BMI (n=450, 19.75%).

**Table 1:** Descriptive statistics of the entire study population. The data has been presented as number of patients (n) and percentages (%).

Characteristic	All	Gout	Pseudogout	No gout
	n (%)	n (%)	n (%)	n (%)
Age group (years), n=4413				
<40	1369 (31.02)	49 (7.53)	0 (0)	1320 (35.51)
40-65	1778 (40.29)	283 (43.47)	7 (15.56)	1488 (40.03)
≥65	1266 (28.69)	319 (49)	38 (84.44)	909 (24.46)
Sex, n=4413				
Female	2509 (56.85)	131 (20.12)	26 (57.78)	2352 (63.28)
Male	1904 (43.15)	520 (79.88)	19 (42.22)	1365 (36.72)
Race/ethnicity, n=3920				
Hispanic or Latino	3356 (85.61)	351 (72.37)	31 (73.81)	2974 (87.65)
Non-Hispanic or non-Latino	564 (14.39)	134 (27.63)	11 (26.19)	419 (12.35)
Marital status, n=3495				
Married/partner	1808 (51.73)	311 (70.36)	25 (71.43)	1472 (48.77)
Single/divorced/widow	1687 (48.27)	131 (29.64)	10 (28.57)	1546 (51.23)
BMI groups, n=2278				
Normal	450 (19.75)	17 (6.72)	4 (10.53)	429 (21.59)
Overweight	608 (26.69)	65 (25.69)	16 (42.11)	527 (26.52)
Obese	1220 (53.56)	171 (67.59)	18 (47.37)	1031 (51.89)

**Table 2:** Descriptive statistics of the subset population. The data has been presented as number of patients (n) and percentages (%).

Characteristic	All (n=1882)	Gout (n=193)	Pseudogout (n=30)	No gout (n=1659)
	n (%)	n (%)	n (%)	n (%)
Age group (years)				
<40	545 (28.96)	17 (8.81)	0 (0)	528 (31.83)
40-65	881 (46.81)	101 (52.33)	5 (16.67)	775 (46.71)
≥65	456 (24.23)	75 (38.86)	25 (83.33)	356 (21.46)
Sex				
Female	1153 (61.26)	36 (18.65)	17 (56.67)	1100 (66.31)
Male	729 (38.74)	157 (81.35)	13 (43.33)	559 (33.69)
Race/ethnicity				
Hispanic or Latino	1591 (84.54)	131 (67.88)	20 (66.67)	1440 (86.8)
Non-Hispanic or non-Latino	291 (15.46)	62 (32.12)	10 (33.33)	219 (13.2)
Marital status				
Married/partner	1080 (57.39)	146 (75.65)	22 (73.33)	912 (54.97)
Single/divorced/widow	802 (42.61)	47 (24.35)	8 (26.67)	747 (45.03)
BMI groups				
Normal	381 (20.24)	10 (5.18)	3 (10)	368 (22.18)
Overweight	512 (27.21)	51 (26.42)	11 (36.67)	450 (27.12)
Obese	989 (52.55)	132 (68.39)	16 (53.33)	841 (50.69)

Factors associated with gout

Based on univariable logistic regression analyses, patients in the age groups 40-65 years and ≥65 years compared to patients aged <40 years old had significantly higher odds of having gout versus no gout (Table 3). Women compared to men had 88% lower odds of having gout versus no gout (OR=0.12; 95% CI: 0.08, 0.17; p<0.0001), as well as 83% lower odds of having gout versus pseudogout (OR=0.17; 95% CI: 0.08, 0.39; p<0.0001) (Table 3). In our sample, Hispanic or Latino individuals compared to non-Hispanic and non-Latino individuals had significantly lower odds of gout (OR=0.32; 95% CI: 0.23, 0.45; p<0.0001) and pseudogout (OR=0.30; 95% CI: 0.14, 0.65; p<0.0001) versus no gout, respectively (Table 3). Additionally, those who were overweight and obese compared to normal-BMI patients had significantly higher odds of having gout compared to patients with no gout (Table 3). These findings remain similar in a multivariable multinomial logistic regression model including age, sex, race/ethnicity, marital status, and BMI groups (Table 4), except that marital status was no longer significantly associated with gout status, adjusting for the rest of the variables included in the model (Table 4).

**Table 3:** Univariable logistic regression analyses for gout status and socio-demographic characteristics. The data has been presented as odds ratios (ORs) with confidence intervals (CIs) of 95% and p-values with statistical significance being p<0.05. N/A: not available

Characteristic	Gout versus no gout			Pseudogout versus no gout			Gout versus pseudogout		
	OR (95% CI)	Wald chi-square value	P-value	OR (95% CI)	Wald chi-square value	P-value	OR (95% CI)	Wald chi-square value	P-value
Age group (years)									
<40	Reference								
40-65	4.05 (2.39, 6.85)	27.18	<0.0001	N/A	0.003	0.9581	N/A	0.002	0.9629
≥65	6.54 (3.80, 11.27)	45.91	<0.0001	N/A	0.004	0.9499	N/A	0.003	0.9564
Sex									
Female	0.12 (0.08, 0.17)	125.42	<0.0001	0.66 (0.32, 1.37)	1.21	0.2657	0.17 (0.08, 0.39)	17.84	<0.0001
Male	Reference			Reference			Reference		
Race/ethnicity									
Hispanic or Latino	0.32 (0.23, 0.45)	44.42	<0.0001	0.30 (0.14, 0.65)	9.13	0.0024	1.07 (0.47, 2.41)	0.02	0.8803
Non-Hispanic or non-Latino	Reference			Reference			Reference		
Marital status									
Married/partner	2.54 (1.81, 3.58)	28.54	<0.0001	2.25 (0.99, 5.09)	3.81	0.0508	1.13 (0.47, 2.71)	0.07	0.7845
Single/divorced/widow	Reference			Reference			Reference		
BMI groups									
Normal	Reference			Reference			Reference		
Overweight	4.17 (2.09, 8.33)	16.37	<0.0001	3.00 (0.83, 1.83)	2.81	0.0937	1.39 (0.33, 5.90)	0.2	0.6546
Obese	5.78 (3.00, 11.11)	25.59	<0.0001	2.31 (0.67, 7.97)	1.8	0.1856	2.49 (0.62, 10.02)	1.63	0.1978

**Table 4:** Multivariable multinomial logistic regression model for gout status and socio-demographic characteristics. The data has been presented as odds ratios (ORs) with confidence intervals (CIs) of 95% and p-values with statistical significance being p<0.05. N/A: not available

Characteristic	Gout versus no gout			Pseudogout versus no gout			Gout versus pseudogout		
	OR (95% CI)	Wald chi-square value	P-value	OR (95% CI)	Wald chi-square value	P-value	OR (95% CI)	Wald chi-square value	P-value
Age group (years)									
<40	Reference			Reference			Reference		
40-65	4.06 (2.24, 7.35)	21.35	<0.0001	N/A	0.003	0.9506	N/A	0.002	0.9637
≥65	4.80 (2.55, 9.04)	23.55	<0.0001	N/A	0.004	0.9588	N/A	0.003	0.956
Sex									
Female	0.10 (0.07, 0.4)	135.36	<0.0001	0.65 (0.30, 1.42)	1.16	0.2807	0.18 (0.08, 0.39)	19.44	<0.0001
Male	Reference			Reference			Reference		
Race/ethnicity									
Hispanic or Latino	0.52 (0.34, 0.78)	9.83	0.0017	0.86 (0.38, 1.94)	0.14	0.7099	0.61 (0.25, 1.46)	1.25	0.2635
Non-Hispanic or non-Latino	Reference			Reference			Reference		
Marital status									
Married/partner	1.18 (0.79, 1.76)	0.63	0.4282	1.23 (0.53, 2.88)	0.23	0.6301	0.96 (0.38, 2.39)	0.01	0.9231
Single/divorced/widow	Reference			Reference			Reference		
BMI groups									
Normal	Reference			Reference			Reference		
Overweight	3.28 (1.58, 6.79)	10.23	0.0014	2.12 (0.57, 7.82)	1.26	0.2617	1.55 (0.36, 6.71)	0.34	0.5572
Obese	5.46 (2.73, 10.92)	23.11	<0.0001	2.24 (0.63, 7.95)	1.56	0.2124	2.44 (0.59, 10.04)	1.53	0.2164

## Discussion

Individuals aged 40-65 and greater than 65 years compared to patients aged less than 40 years old had significantly higher odds of having gout versus no gout in our study. Women compared to men had 88% lower odds of having gout compared to no gout and 83% lower odds of having gout compared to pseudogout in our study. The current literature agrees with our study as it states that increased age and men have an increased frequency of gout compared to younger individuals and women, both in the United States and globally [1,3,4]. Also of note, gout frequency is significantly higher in men compared to women in Western nations such as the United States, which also agrees with our results as men had 88% higher odds of having gout, a very significant difference [1]. In regard to pseudogout, some literature states that men are more likely than women to have pseudogout, whereas other literature states that men and women have an equal frequency of pseudogout [5,12]. However, we were unable to find any studies comparing gout to pseudogout in terms of odds of obtaining these conditions based on sex as we did in this study.

Hispanic or Latino ethnicity, compared to non-Hispanic and non-Latino ethnicity, had significantly lower odds of gout and pseudogout compared to no gout in our study, indicating a possible protective effect of Hispanic/Latino ethnicity against these conditions. In the past, gout studies have focused on White and Black adults; however, recent literature has been published reviewing various other ethnicities [13]. This literature states that although Native Hawaiian, Black, and Japanese individuals in the study had a higher risk of incident gout compared to White participants, Hispanic or Latino individuals had a lower risk [13]. This agrees with our study results, although the exact reason behind this trend is unclear. It may possibly be due to a difference between ethnicities in various lifestyle factors that are associated with gout and pseudogout, such as the consumption of red meats, alcohol, and seafood [1,2]. Regarding pseudogout, to our knowledge, there are no studies comparing differences between various ethnicities, specifically the Hispanic or Latino population to other ethnicities.

Individuals classified as overweight (OR {95% CI}: 3.28 {1.58, 6.79}) or obese (OR {95% CI}: 5.46 {2.73, 10.92}) compared to normal-BMI patients had significantly higher odds of having gout compared to patients with no gout in our study. The current literature shows that in the United States, the crude prevalence of gout is around 1%-2% among individuals with a normal BMI (18.5-24.9 kg/m^2^), 3% among overweight individuals, 4%-5% in individuals classified as class I obesity, and 5%-7% in individuals classified as class II or class III obesity [14]. This agrees with our study results as there was a progressively higher prevalence ratio of gout associated with successively higher categories of BMI [14].

Marital status was the only variable that showed a difference between univariable and multivariable analysis and was not significantly associated with gout status according to the multivariable model. To our knowledge, there have been no studies conducted on the frequency of gout in individuals of different marital statuses.

The limitations of this study are as follows. Data was collected solely from UTRGV UTHealth electronic databases, so individuals who received care at a non-UTRGV-affiliated institution were not taken into account in this study. Therefore, this study may not be generalizable to the South Texas population as a whole. Also, the majority of the patients analyzed in this study were Hispanic individuals, which indicates that our results may not be generalizable to another population whose ethnic landscape differs from our study. Lastly, the RGV is an impoverished, medically challenged area with numerous undocumented immigrants and individuals without health insurance [9,10]. This patient community is another reason that the results of this study may not be universally applied. Future studies should focus on obtaining data from a larger, more generalizable source and consider including more patient demographic factors and characteristics that could play a role in the development of gout and pseudogout to increase study validity and generalizability.

## Conclusions

Individuals of various demographics in the underserved RGV community, and possibly demographically similar communities, may be at increased risk for gout or pseudogout. This warrants further research on these conditions in this region to further improve the knowledge and possibly prevent numerous effects of these conditions on an individual’s quality of life.
